# Radiation Response of Human Cardiac Endothelial Cells Reveals a Central Role of the cGAS-STING Pathway in the Development of Inflammation

**DOI:** 10.3390/proteomes8040030

**Published:** 2020-10-26

**Authors:** Jos Philipp, Ronan Le Gleut, Christine von Toerne, Prabal Subedi, Omid Azimzadeh, Michael J. Atkinson, Soile Tapio

**Affiliations:** 1 Institute of Radiation Biology, Helmholtz Center Munich, German Research Center for Environmental Health GmbH, 85764 Neuherberg, Germany; jos.philipp@rub.de (J.P.); psubedi@bfs.de (P.S.); omid.azimzadeh@helmholtz-muenchen.de (O.A.); atkinson@helmholtz-muenchen.de (M.J.A.); 2 Institute of Computational Biology, Helmholtz Center Munich, German Research Center for Environmental Health GmbH, 85764 Neuherberg, Germany; ronan.legleut@helmholtz-muenchen.de; 3 Research Unit Protein Science, Helmholtz Center Munich, German Research Center for Environmental Health GmbH, 85764 Neuherberg, Germany; vontoerne@helmholtz-muenchen.de; 4 Federal Office for Radiation Protection, BfS, 85764 Neuherberg, Germany; 5 Chair of Radiation Biology, Technical University of Munich, 80333 Munich, Germany

**Keywords:** ionizing radiation, proteomics, inflammation, cGAS-STING-pathway, DDB2, endothelial cells, STAT1, data-independent acquisition

## Abstract

Radiation-induced inflammation leading to the permeability of the endothelial barrier may increase the risk of cardiovascular disease. The aim of this study was to investigate potential mechanisms in vitro at the level of the proteome in human coronary artery endothelial cells (HCECest2) that were exposed to radiation doses of 0, 0.25, 0.5, 2.0 and 10 Gy (60Co-γ). Proteomics analysis was performed using mass spectrometry in a label-free data-independent acquisition mode. The data were validated using bioinformatics and immunoblotting. The low- and moderate-dose-irradiated samples (0.25 Gy, 0.5 Gy) showed only scarce proteome changes. In contrast, an activation of DNA-damage repair, inflammation, and oxidative stress pathways was seen after the high-dose treatments (2 and 10 Gy). The level of the DNA damage response protein DDB2 was enhanced early at the 10 Gy dose. The expression of proteins belonging to the inflammatory response or cGAS-STING pathway (STING, STAT1, ICAM1, ISG15) increased in a dose-dependent manner, showing the strongest effects at 10 Gy after one week. This study suggests a connection between the radiation-induced DNA damage and the induction of inflammation which supports the inhibition of the cGAS-STING pathway in the prevention of radiation-induced cardiovascular disease.

## 1. Introduction

Endothelial cells form the inner single-cell layer surrounding every blood vessel, thus representing the barrier between blood and the tissues underneath. Endothelial cells are responsible for taking up nutrients and delivering waste products to the blood, thereby protecting the tissue, communicating with immune cells and controlling the blood flow [1]. Signal mechanisms induced by external stressors such as ionizing radiation, chemicals or pathogenic agents trigger an acute inflammatory immune response that functions in a paracrine, endocrine and/or autocrine manner [2,3,4,5]. The initial signaling cascades include the activation of endothelial signaling molecules like nitric oxide or adhesion molecules but also inflammatory proteins. In the case of ionizing radiation, both the radiation dose and the time elapsing after the exposure influence the inflammatory outcome [6,7]. In the worst case, the initial acute inflammation may progress to a chronic one [8,9,10,11]. Sustained low-level inflammation is a risk factor for cardiovascular disease by promoting atherosclerotic plaque formation, rupture and finally thrombosis [12].

High-throughput “omics” technologies such as proteomics enable a deeper understanding of biological processes on different levels and scales [13]. This has the advantage to display not only single pathways but to observe changes in several pathways simultaneously within one measurement. In radiation biology, dose- and time-dependent measurements are necessary to reveal changes in the proteome leading to morphological and functional alterations at a cellular level. In endothelial cells, due to their complex barrier forming system, it is particularly relevant to investigate whole processes taking place after radiation exposure.

In endothelial cells, low and moderate doses (<0.5 Gy) may attenuate an ongoing inflammatory process [14,15]. If the cells are not in an inflammatory state at the time of irradiation, both pro- and anti-inflammatory effects or no radiation influence on inflammatory parameters have been reported [16,17,18,19] whereas high doses are clearly pro-inflammatory [20,21,22,23]. At high doses (>0.5 Gy), endothelial cells express and release cytokines such as IL-6, IL-8, MCP1, and type I interferon-related proteins to attract immune cells [24,25]. In addition, other pro-inflammatory proteins, especially intracellular and vascular adhesion molecules like ICAM1, VCAM1 and PECAM1 are increasingly present at the cell surface [26]. This leads to the attachment and infiltration of immune cells into the surrounding tissue and later on to senescence [23].

DNA damage caused by radiation exposure results in the accumulation of cytosolic DNA that is recognized by the cGAS-STING pathway [27,28] alerting the cell’s immune system [29,30]. In a complex cascade, cGAS generates the second messenger cGAMP that activates the adaptor protein STING which, in its turn, activates the transcription factor IRF3 in a phosphorylation-dependent mechanism [31]. This triggers the induction of type I interferon-related pro-inflammatory immune response [32]. Recent research has provided strong evidence that cGAS also has an essential role in promoting cellular senescence via the senescence-associated secretory phenotype, SASP [2,24,33], thereby connecting DNA damage, inflammation and senescence [30,34].

Previously, we reported a pro-inflammatory radiation response in human coronary artery endothelial cell line (HCECest2) and primary murine lung endothelial cells after a dose of 10 Gy X-ray [24,34]. In both cases, a strong inflammatory reaction based on the activation of signal transducer and the activator of transcription 1 (STAT1), a transcription factor that induces the expression of interferon (IFN)-related and other inflammatory genes, was observed [35,36]. Consequently, a strong upregulation of ubiquitin-like protein ISG15 was observed in both studies where the pro-inflammatory phenotype appeared relatively late in the in vitro conditions, 14 and 10 days after the radiation exposure in the two studies, respectively.

Based on our previous results and the fact that STAT1 is known to be activated by the cGAS-STING pathway [37], we hypothesized that this pathway could be activated by ionizing radiation in endothelial cells from early on to trigger the pro-inflammatory response. However, we preferred to look at the global proteome changes rather than only some selected proteins not to miss any previously less defined components of the inflammatory pathway. Furthermore, the proteomic approach used in this study could work as a data source for several future publications. In this study, however, we focused on a continuation of our previous work examining the associated proteins of the cGAS-STING pathway, a task that can hardly be performed using immunoblotting only. Therefore, we investigated the dose- and time-dependent alterations in the endothelial proteome globally but with a particular focus on the cGAS-STING pathway. This systematic analysis using the newest proteomics methods was necessary since the initiation and progression of radiation-induced inflammatory response in endothelial cells is poorly understood. Gaining more information about the inflammatory processes in endothelial cells could open the door for new possibilities in the prevention and alleviation of radiation-associated cardiovascular disease.

## 2. Materials and Methods

### 2.1. Cell Culture and Irradiation

**Figure 1:**
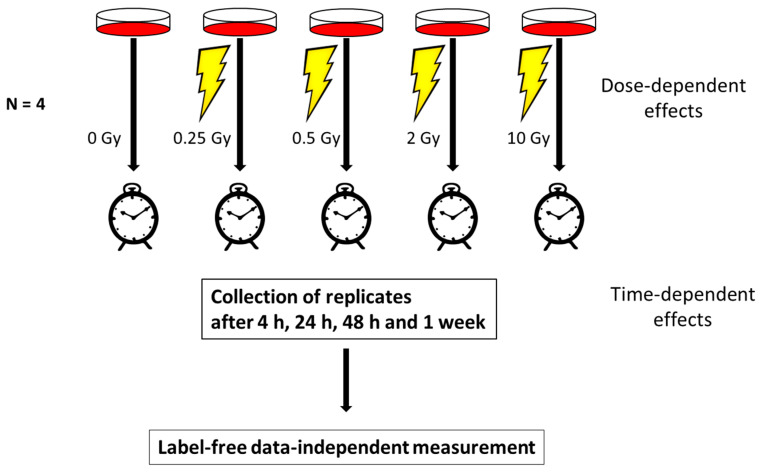
The work flow of the study showing the experimental design. Human coronary artery endothelial cells (HCAECs) were irradiated with 0, 0.25, 0.5, 2 or 10 Gy. At 4, 24, 48 h and 1 week, the cells were harvested and analyzed using a data-independent proteomics approach. The proteome changes in all irradiated samples were normalized to the sham-irradiated sample collected at the corresponding time point to investigate the dose-dependent effects. The proteome changes at different time points were normalized to the corresponding sample collected at 4 h.

Human telomerase-immortalized coronary artery endothelial cells (HCECest2) obtained from Dr. Ken Raj, were cultured at 37 °C and 5% CO_2_ as described previously [23]. Cells were grown in Human MesoEndo Cell Growth Medium Kit (Cell Applications, Inc., San Diego, CA, USA) until confluency. Cells were irradiated with a Caesium-137 source (HWM-D-2000, dose rate 400 mGy/min) with γ-doses of 0 Gy (sham irradiation), 0.25, 0.5, 2 and 10 Gy and were harvested after 4, 24, 48 h and one week. During the experiment, the cells were not passaged but every two days a new medium was applied. Time- and radiation-dependent cell morphology changes were recorded by taking microscopic images using Keyence BZ-9000 (Keyence Corporation, Neu-Isenburg, Germany) with a 4×/0.20 objective. The cells were harvested by removing the medium, washing twice with ice-cold phosphate buffered saline, scraping and centrifugation at 300× *g* for 5 min. The supernatant was discarded and the cell pellets frozen at −80 °C until further use. Four biological replicates of each time and dose point were made. The replicates were cultivated at different time points. The work flow is shown in Figure 1.

### 2.2. Cell Lysis and Sample Preparation

All cell pellets were lysed with RIPA buffer (Thermo Fisher Scientific, Schwerte, Germany) containing phosphatase inhibitor (PhosStop, Roche Applied Science, Penzberg, Germany) and protease inhibitor (cOmpleteTM, Roche, Darmstadt, Germany) following the manufacturer’s instructions. Protein concentration was determined using the PierceTM BCA Protein Assay Kit (Thermo Fisher Scientific) according to the manufacturer’s instructions. Sample preparation was done using FASP digest [38].

### 2.3. Mass Spectrometry (MS)

MS data were acquired in data-independent acquisition (DIA) mode on a Q Exactive (QE) high field (HF) mass spectrometer (Thermo Fisher Scientific) as described previously [39] with the following changes: peptides were eluted from column at 250 nl/min using an increasing acetonitrile (ACN) concentration (in 0.1% formic acid) from 3% to 41% over a 105 min gradient. Precursor peptides were isolated with 37 variable windows spanning from 300 to 1650 *m*/*z* at 30,000 resolution with an AGC target of 3e6 and automatic injection time.

### 2.4. Spectral Library, Spectronaut Analysis and Data Processing

Selected LC–MS/MS DDA data encompassing 80 raw files were analyzed using Proteome Discoverer (Version 2.1, Thermo Fisher Scientific) using Byonic (Version 2.0, Proteinmetrics, San Carlos, CA, USA) search engine node maintaining 1% peptide and protein false discovery rate (FDR) threshold. The peptide spectral library was generated in Spectronaut (Version 10, Biognosys, Schlieren, Switzerland) with default settings using the Proteome Discoverer result file. Spectronaut was equipped with the SwissProt human database (Release 2017.02, 20,194 sequences, www.uniprot.org) with a few spiked proteins (e.g., Biognosys iRT peptide sequences). The final spectral library generated in Spectronaut contained 11,505 protein groups and 417,843 peptide precursors. Mastermix containing peptides from all treatments was used to obtain the same quality in all measurements. The DIA MS data were analyzed using the Spectronaut 12 software as described previously [34].

### 2.5. Statistical Analysis

For the statistical analysis, the R software (version 3.52.0, GNU General Public License, Boston, MA, USA) was used. The “normalized” abundances were filtered for proteins identified with more than one unique peptide. The vsn R package (version 3.52.0) [40] was then used for an affine transformation and a generalized log2 transformation of the protein expression. Two-dimension reduction techniques, UMAP [41] and t-SNE [42], were then applied on the transformed data, which helped us to identify a strong batch effect due to the 4 replicates. The differential expression analysis was run using the R package LIMMA (version 3.40.2) [43] using a generalized linear mixed model including the interaction between the different doses and time points, as fixed effects and the replicates as a random effect to account for the similarities within the replicates. The significance of protein deregulation was based on the label-free proteomics analysis (fold change of ±1.3; Storey-corrected *p*-value q < 0.05 [44]; *n* = 4) and immunoblotting (*t*-test; *p* < 0.05; *n* = 3).

### 2.6. Bioinformatics

Proteins were grouped based on the time and dose. Grouped proteins were further investigated for pathway affiliation using ingenuity pathway analysis (IPA, Qiagen, Inc., Hilden, Germany, https://www.qiagenbioinformatics.com/products/ingenuitypathway-analysis).

### 2.7. Immunoblotting

Immunoblotting was done as described previously [35]. The following antibodies were purchased from Merck Millipore: cGAS (ABF124) and from Santa Cruz (Santa Cruz Biotechnology, Inc., Heidelberg, Germany): STAT1 (sc-346), ISG15 (sc-166755), ICAM1 (sc-8435).

### 2.8. Data Availability

The MS proteomics data were deposited to the ProteomeXchange Consortium via the PRIDE [45] partner repository with the dataset identifier PXD020735.

## 3. Results

### 3.1. Radiation Dose and Time Effects on the Proteome

Proteome analysis of the irradiated cells at different time points and irradiation doses was performed label-free and in a data-independent acquisition mode to also identify low abundant proteins. In total, 4060 proteins were identified. The dataset was statistically analyzed for the radiation dose (Tables S1–S4) and time (Tables S6–S10).

**Figure 2:**
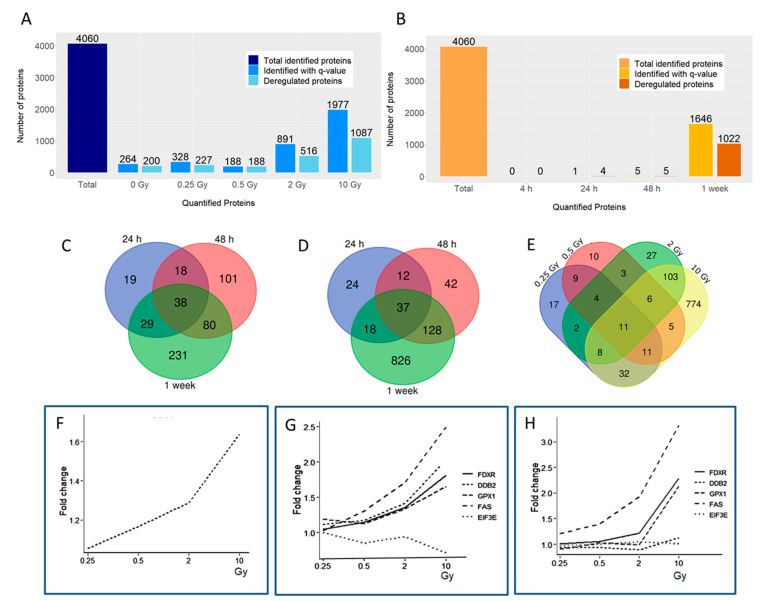
The alterations in the human coronary artery endothelial cell line (HCECest2) cell proteome with time and radiation dose. (**A**) The total number of all identified proteins (dark blue), the number of all quantified (q ≤ 0.05) proteins (blue) and the number of significantly differentially regulated (q ≤ 0.05; fold change ±1.3) proteins (light blue) at each time point compared to the 4 h time point are shown. (**B**) The total number of all identified proteins (light orange), the number of all quantified (q ≤ 0.05) proteins (orange) and the number of significantly differentially regulated (q ≤ 0.05; fold change ±1.3) proteins (dark orange) at each radiation dose compared to the 0 Gy control are shown. (**C**) Venn diagram illustrating the number of shared deregulated proteins between the three time points at 2 Gy. (**D**) Venn diagram illustrating the number of shared deregulated proteins between the three time points at 10 Gy. (**E**) Venn diagram illustrating the number of shared deregulated proteins between the four radiation doses at the 1 week time point. (**F**) The level of DDB2 (DNA damage-binding protein 2) at different radiation doses 24 h post-exposure is shown. (**G**) The level of deregulated proteins FDXR (ferredoxin reductase), DDB2, GPX1 (glutathione peroxidase 1), FAS (tumor necrosis factor receptor superfamily member 6), and EIF3 (eukaryotic translation initiation factor 3 subunit E) at different radiation doses 48 h post-exposure is shown. (**H**) The level of deregulated proteins FDXR, DDB2, GPX1, FAS, and EIF3 at different radiation doses 1 week post-exposure is shown.

Time-dependent effects were measured against the 4 h time point, the first time point with proteomics data. A large number of proteins were found to be deregulated in a time-dependent manner, especially at 2 and 10 Gy, with 516 and 1087 deregulated proteins, respectively (Figure 2A). In addition, 227 and 188 deregulated proteins were found for the 0.25 and 0.5 Gy dose points, respectively.

The radiation, in contrast, only marginally affected the protein expression at the early time points (Figure 2B). No proteins were deregulated at 4 h post-radiation compared to the control (0 Gy). Similarly, the number of deregulated proteins 24 and 48 h post-radiation, being one and five proteins, respectively, was low (Figure 2B). Nevertheless, a strong radiation effect with 1022 deregulated proteins at all doses was observed after one week (Figure 2B). At 10 Gy, 950 proteins were deregulated using the fold change of ± 1.3 that we then used for all time points and radiation doses. Increasing the fold change to ±1.5 or ±2.0 decreased the number of deregulated proteins at 10 Gy to 413 and 67, respectively.

The clear increase in the number of deregulated proteins in a time-dependent manner is illustrated in the Venn diagrams at 2 (Figure 2C) and at 10 Gy (Figure 2D). The Venn diagram illustrating the number of shared deregulated proteins between the four radiation doses was shown in Figure 2E. The number of deregulated proteins changed with the radiation dose as follows: 94 (5.7% of all quantified proteins) were deregulated at 0.25 Gy, 59 (3.6%) at 0.5 Gy, 164 (10.0%) at 2 Gy, and 950 (57.7%) proteins at 10 Gy. Only 11 proteins were deregulated at every radiation dose. Most shared proteins (128) were found between the two higher doses (Figure 2E).

Only one protein, DNA damage-binding protein 2 (DDB2), was significantly deregulated (2, 10 Gy) at 24 h showing upregulation (Figure 2F). It was also upregulated at 48 h (10 Gy), similar to the proteins ferredoxin reductase (FDXR), glutathione peroxidase 1 (GPX1), and tumor necrosis factor receptor superfamily member 6 (FAS) (Figure 2G). The only downregulated protein at 48 h was the elongation factor EIF3E (10 Gy). After one week, DDB2 and EIF3E were no longer deregulated but the proteins FDXR, GPX1 and FAS all remained upregulated (Figure 2H). When clustering the proteins deregulated after one week, most proteins at the lower doses of 0.25 and 0.5 Gy showed a similar direction of deregulation that was inverted at the higher doses of 2 and 10 Gy (Figure S1). In addition, the deregulated proteins at 0.25 and 0.5 Gy clustered together as did those at 2 and 10 Gy (Figure S1).

The ingenuity pathway analysis (IPA) showed that the most important altered molecular function as a function of the radiation dose was the inflammatory response (Table S5). The first three functional categories with the lowest *p*-value and the highest number of proteins all include the term inflammation (Table S5).

### 3.2. The Expression of Proteins Involved in DNA Repair, Oxidative Stress and Inflammatory Response

To further analyze the radiation-affected pathways, the expressions of proteins involved in DNA repair, inflammatory response, and oxidative stress was investigated in more detail.

**Figure 3:**
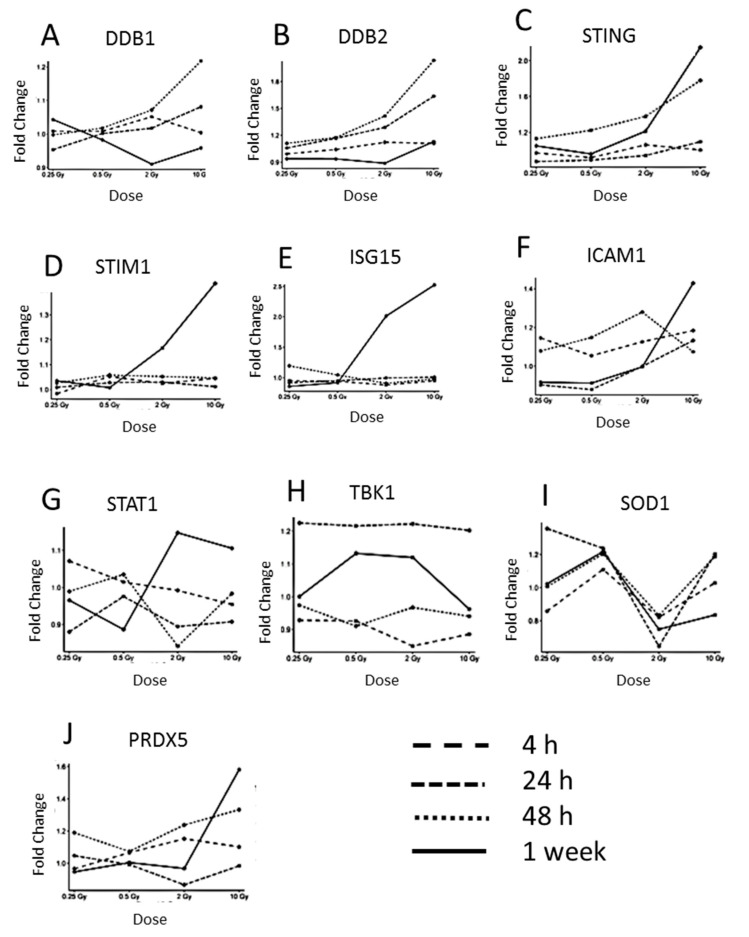
The dose-dependent alteration in the expression of proteins belonging to functionally different groups: DNA repair pathway, inflammatory response, and oxidative stress. (**A**) The level of DDB1 (DNA damage-binding protein 1), (**B**) DDB2 (DNA damage-binding protein 2), (**C**) STING (stimulator of interferon genes protein), (**D**) STIM1 (stromal interaction molecule 1), (**E**) ISG15 (ubiquitin-like protein ISG15), (**F**) ICAM1 (intercellular adhesion molecule 1), (**G**) STAT1 (signal transducer and activator of transcription 1), (**H**) TBK1 (serine/threonine-protein kinase TBK1), (**I**) SOD1 (superoxide dismutase (Cu–Zn)), and (**J**) PRDX5 (peroxiredoxin-5, mitochondrial) is shown after 4, 24, 48 h, and 1 week.

The expression of the DNA-repair proteins DDB1 and DDB2 was upregulated at the highest dose of 10 Gy at 24 and 48 h time point, then declining after one week (Figure 3A,B), indicating radiation-induced DNA damage.

Then, we investigated the level of proteins of the cGAS-STING-pathway. As the cGAS protein was not present in the proteomics data, probably due to its low abundance, the expression of its downstream targets was examined. Additionally, alterations in the protein expression associated with the type I interferon response that are known to be induced by the upregulation of STING [46] were studied. The expression of STING was markedly upregulated at 10 Gy from the 48 h time point onwards (Figure 3C). The STIM1 protein that is known to co-localize with STING in the endoplasmic reticulum was also upregulated at 2 and 10 Gy after one week (Figure 3D). As previously shown, the type I interferon response in endothelial cells is activated by irradiation [24,35]. Similar to our previous studies, the expression of ISG15, a type I interferon-related protein, was highly upregulated after 2 and 10 Gy at the 1 week time point (Figure 3E). In a similar fashion, the level of ICAM1 was also upregulated after 1 week at the dose of 10 Gy (Figure 3F). STAT1, a key player of the type I Interferon response, was upregulated at 2 and 10 Gy 1 week post-radiation (Figure 3G). In contrast, the expression of the cGAS downstream target TBK1 did not change significantly (Figure 3H). Additional inflammatory proteins such as interferon-induced GTP-binding proteins MX1 and MX2, proteins of the oligoadenylate synthetase (OAS) family and proteins of the IFIT family that are known to be induced in response to the type I Interferon-related activation, did not show significant time- or dose-dependent expression changes (Figure S2).

Inflammation is linked to increased oxidative stress in cardiovascular disease [47]. In order to monitor the possible changes in the oxidative stress response, the levels of superoxide dismutase 1 (SOD1) and peroxiredoxin 5 (PRDX5) were investigated (Figure 3I,J). The expression of SOD1 was not deregulated in a dose-dependent manner. In contrast, PRDX5 was upregulated after 1 week at the dose of 10 Gy.

In addition to the DNA repair, inflammatory and oxidative stress proteins, we found a large number of mitochondrial proteins differentially regulated, especially members of the respiratory complex I 1 week post-radiation (Tables S4 and S5). These, as well as mitochondrial antiviral-signaling protein (MAVS), were upregulated emphasizing the role of mitochondria in the endothelial radiation response.

### 3.3. Immunoblotting Validation 1 Week Post-Radiation

**Figure 4:**
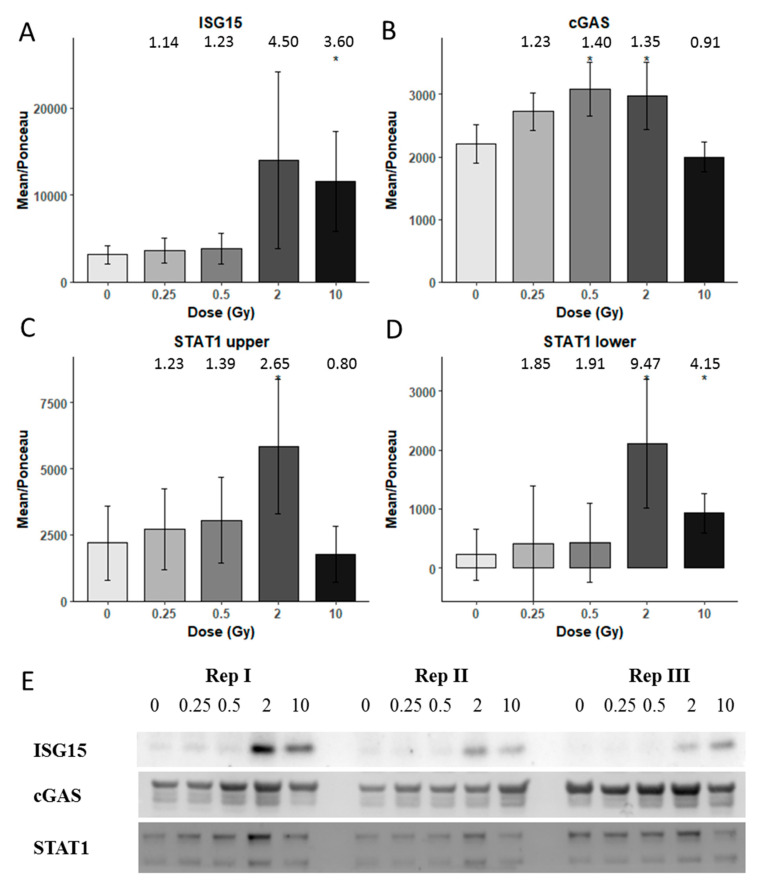
Immunoblot verification of the protein changes in the irradiated HCECest2 cells 1 week after radiation exposure. (**A**) The expression of ISG15 (ubiquitin-like protein ISG15), (**B**) cGAS (cyclic GMP-AMP synthase), (**C**) STAT1 (signal transducer and activator of transcription 1) upper band, (**D**) STAT1 lower band is shown. The bars represent the relative expression after correction for background and normalization to Ponceau. The error bars are calculated as the SEM (*t* test; * *p* < 0.05; *n* = 3). (**E**) The visualization of protein bands is shown.

Methodological validation of the proteomics data was performed using immunoblotting (Figure 4). Proteins involved in the cGAS-STING pathway or inflammatory response were tested. After one week, a significant upregulation for ISG15 was found at 10 Gy (Figure 4A), whereas cGAS was significantly upregulated at 0.5 and 2 Gy, but interestingly, not at 10 Gy (Figure 4B). STAT1 showed two bands in the blotting. The upper band was significantly upregulated only at 2 Gy but the lower band showed upregulation both at 2 and 10 Gy (Figure 4C,D), in accordance with the proteomics data. The immunoblots are shown in Figure 4E.

The time-dependent expression changes of ICAM1 were tested with immunoblotting (Figure S3). The level of ICAM1 was increased after one week independently of the radiation dose and even in the control.

## 4. Discussion

In the standard radiation therapy for treating breast cancer, the applied dose varies from 0.1 to 20 Gy in the left anterior descending artery [48], and a similar average dose exposure to the lung was reported [49]. Considering the increasing risk for vascular and cardiac disease in breast cancer survivors, it is important to investigate the endothelial response to a range of different radiation doses [50,51]. In this study, we investigated a dose- and time-related response of endothelial cells after irradiation with doses ranging from 0.25 to 10 Gy over one week. These doses correspond to the cardiac doses received in radiation therapy, depending on the location of the tumor.

Previously, we saw the induction of pro-inflammatory proteins two weeks post-radiation in HCECest2 cells [24] and one week post-radiation in primary mouse lung endothelial cells [35]; both studies used a radiation dose of 10 Gy. In addition, long-term studies (16 weeks) performed in cardiac endothelial cells isolated from mice exposed to local heart radiation (16 Gy) revealed an induction of inflammation by assessing global proteome changes or selected surface markers [9,10]. Using the same cell line as in this study, the increased adhesion of monocytes and the release of pro-inflammatory cytokines (interleukins 6 and 8) was shown already at the dose of 2 Gy X-rays [52]. This effect became significant seven days after the exposure. At lower radiation doses, the induction of inflammatory response is contradictory [16,17,18,19], although significant changes in other processes such as NO signaling or protein degradation have been observed already at the dose of 0.5 Gy in vitro, especially after 14 days [17].

These previous studies are in agreement with the data presented here. The doses of 0.25 Gy and 0.5 Gy triggered some proteomic changes after one week but these alterations did not contribute to the inflammatory response. Interestingly, the results from cellular studies are in accordance with the epidemiological data suggesting a threshold dose for the induction of radiation-induced cardiovascular disease of about 0.5 Gy [53,54] with only a scarce number of studies showing an increased risk at doses slightly lower than that [55,56].

At 24 and 48 h, the 2 and 10 Gy doses increased the expression of DDB2 significantly, indicating an induction of the DNA repair process. DDB2 is one of the best radiation biomarkers not only in the human blood but also in different types of cells [57,58,59,60]. However, to the best of our knowledge, this is the first study to find a radiation-associated increase in DDB2 in endothelial cells. DDB2 is regulated by p38MAPK and p53 [61,62]. It was found to be one of the 16 blood biomarkers of aging revealing a role not only in the radiation response but also in the pathology of senescence [63].

Also indicating DNA damage is the dose-dependent upregulation of the FDXR protein, also known as ferredoxin reductase. As DDB2, it has been shown to be highly upregulated by ionizing radiation at the transcriptional level, making it one of the most reliable radiation biomarkers in the human blood [64,65,66,67]. This p53-regulated flavoprotein, the first enzyme in the mitochondrial P450 system, also responds also to other DNA-damaging agents rather than ionizing radiation but to a lesser extent [68].

It is well known that ionizing radiation and other DNA-damaging agents are able to induce the expression of type I interferons and other cytokines [69,70,71,72,73,74,75]. A relatively new observation is the essential role of the cGAS-STING pathway in this process, even in non-cancerous cells and tissues [24,35,76]. Our present study showed that cGAS was markedly upregulated at 2 Gy one week post-radiation. At 10 Gy, in contrast to STING which was upregulated about two-fold, the level of cGAS was not changed. Although this result was somewhat surprising, it supported our previous data with primary lung endothelial cells where no cGAS upregulation was seen at 10 Gy [35]. In addition to STING, also its counterpart STIM1 that interacts with STING by inactivating it [46] was upregulated at 10 Gy one week after irradiation, indicating a possible negative feedback loop to regulate the STING level. Furthermore, the MAVS protein was significantly upregulated at 2 and 10 Gy one week post-radiation. Similar to STING, MAVS has recently been shown to be necessary for radiation-induced Type I interferon signaling [77]. Then, STING forms a complex with TBK1 allowing its activation by autophosphorylation [78]. This type of activation may explain why we see no changes in TBK1 protein level. TBK1 phosphorylates interferon regulatory transcription factor 3 (IRF3). Phosphorylated IRF3 dissociates from its adapter proteins and in a dimerized form enters the nucleus to induce the expression of interferons [32,79].

**Figure 5:**
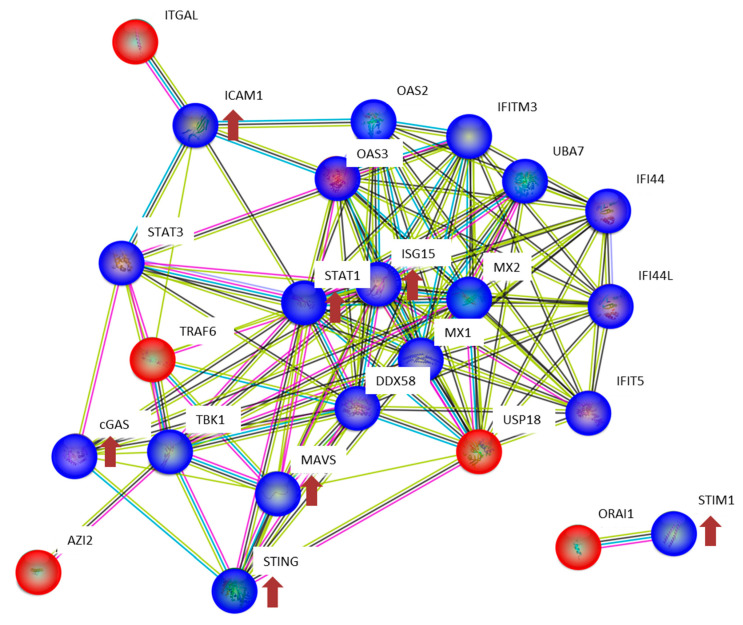
Interaction analysis of the cGAS-STING network using the STRING-db (string-db.org). Direct interactions between the proteins identified in HCECest2 cells one week after irradiation using proteomics or immunoblotting are shown in blue, predicted interactions (one step relaxation in STRING-db) are shown in red. Significantly differentially regulated proteins and the direction of deregulation are shown by red arrows. The significance of deregulation was based on the label-free proteomics analysis (fold change of ±1.3; q < 0.05; n = 4) and immunoblotting (*t*-test; *p* < 0.05; n = 3). The lines between the proteins have the following color code: the red line indicates the presence of fusion evidence, the green line indicates neighborhood evidence, blue line indicates co-occurrence evidence, the purple line indicates experimental evidence, the yellow line indicates text mining evidence, the light blue indicates line database evidence, and the black line indicates co-expression evidence. The abbreviations and full names of the network proteins are as follows: AZI2: 5-azacytidine-induced protein 2; cGAS: cyclic GMP–AMP synthase; DDX58: antiviral innate immune response receptor RIG-I; ICAM1: intercellular adhesion molecule 1; IFI44: interferon-induced protein 44; IFI44L: interferon-induced protein 44-like; IFIT5: interferon-induced protein with tetratricopeptide repeats 5; IFITM3: interferon-induced transmembrane protein 3; ISG15: ubiquitin-like protein ISG15; ITGAL: integrin alpha-L; MAVS: mitochondrial antiviral-signaling protein; MX1: interferon-induced GTP-binding protein Mx1; MX2: interferon-induced GTP-binding protein Mx2; OAS2: 2′-5′-oligoadenylate synthase 2; OAS3: 2′-5′-oligoadenylate synthase 3; ORAI1: calcium release-activated calcium channel protein 1; STAT1: signal transducer and activator of transcription 1-alpha/beta; STAT3: signal transducer and activator of transcription 3; STING: stimulator of interferon genes protein; STIM1: stromal interaction molecule 1; TBK1: serine/threonine-protein kinase TBK1; TRAF6: TNF receptor-associated factor 6; UBA7: ubiquitin-like modifier-activating enzyme 7; USP18: Ubl carboxyl-terminal hydrolase 18.

One of the target proteins of the type I Interferon response is ISG15, which we found to be significantly upregulated after one week (2 and 10 Gy) in agreement with our previous data [24,35]. ISG15 causes ISGylation, a process yet incompletely understood. However, ISGylation has been shown to increase the stability of proteins such as STAT1, preventing a termination of the inflammatory response [80]. Accordingly, the level of STAT1 was significantly increased in the 2 and 10 Gy-treated samples after one week. As shown by us and by others, the ISG15 protein can function as a secreted cytokine allowing the spreading and maintenance of the pro-inflammatory and senescent phenotype [24,81]. The interaction network of the proteins in the cGAS-STING pathway is illustrated in Figure 5.

Interestingly, a large number of proteins changed their expression with time. It has been shown previously that culture conditions strongly influence the expression pattern of several inflammatory proteins increasing the levels of cytokines but decreasing the level of endothelial nitric oxide synthase (eNOS) the function of which is necessary for the vascular tone [82,83,84]. Proteins such as ICAM1, STAT1, and STING were upregulated in the time course of this experiment. However, time-dependent changes were not totally independent of the radiation dose since we found most such alterations in the irradiated cells. Both of these factors, the time and the radiation dose, seem to be necessary for the development of inflammatory response in cardiac endothelial cells.

## 5. Conclusions

This systematic investigation using the newest proteomics technology provides insights in the molecular changes in endothelial cells after the exposure to a range of radiation doses. This data set contains such a large amount of information that it cannot be all included in one study. Based on our previous results, we followed here particular pathways that are known to be induced by irradiation or characteristic for cardiovascular disease, namely inflammatory response, DNA damage, and oxidative stress. The pro-inflammatory state was seen at 2 and 10 Gy as an activation of the cGAS-STING pathway, especially one week after the radiation exposure. At the lower doses of 0.25 and 0.5 Gy, no radiation-induced inflammation could be observed. This study supports the recent effort to develop inhibitors of the cGAS-STING pathway as anti-inflammatory agents [85] that could be used in the prevention and alleviation of radiation-induced cardiovascular disease.

### Limitations of the Study

In this study, we used only one dose rate, namely 400 mGy/min. This relatively low dose rate allowed us to give small doses such as 0.25 Gy accurately. In addition, irradiating the cells with the total dose of 10 Gy took 25 min, which was still possible within the time frame of the experiment. However, we cannot exclude possible additional effects on the proteome if we had used a different dose rate.

The large SEM values that we discovered when using immunoblotting may be due to the fact that we used real biological replicates, meaning that the cells between the different replicates were cultivated at different time points. The cells were irradiated as a confluent monolayer as described before [24] and although all cell cultures were initiated using the same cell number, they may have been at slightly different stages at the time of irradiation.

Biological variation is obviously a limitation of the cell culture model that we used here. However, in order to investigate the large number of doses and time points as in this study, it would have been difficult to use an animal model. To deal with the limitations and exclude a possible effect of contaminants, we followed the cell cultures irradiated with different radiation doses at different time points by taking microscope images (Figure S6). Although we found time-dependent proteome changes even in the sham-irradiated controls, we could not observe any morphological changes Figure S6). The dose of 10 Gy induced the most marked morphological changes: The cell density started to decrease at the same time as the cell size turned bigger (24 h post-irradiation). In addition, the cobblestone monolayer pattern, typical for endothelial cell cultures and as seen in the sham-irradiated, 0.25 and 0.5 Gy irradiated cells, was lost beginning after 48 h at 10 Gy. At day five, the cell density of the 2 Gy irradiated cells also started to decrease.

Although we observe changes in the proteome that indicate DNA damage and inflammatory response after the high-dose radiation, we did not measure these end points directly. The protein changes can only suggest this being the case which is a limitation of this study.

## Supplementary Materials

The following are available online at https://www.mdpi.com/2227-7382/8/4/30/s1, Figure S1: Heat map of all significantly deregulated (1022) proteins in HCECest2 cell line 1 week after different radiation doses; Figure S2: Expression of inflammatory proteins as a function of radiation dose or time; Figure S3: Ponceau staining as the loading control of the immunoblot analysis for Figure 4; Figure S4: Immunoblot verification of ICAM1 levels as a function of time in HCECest2 cells; Figure S5: Ponceau stainings as the loading controls for the immunoblot analysis for Figure S4; Figure S6: Radiation-induced cell morphology changes of the endothelial cell line HCECest2; Table S1: All the identified, quantified, and deregulated proteins at different radiation doses compared to the sham-irradiated control at 4 h; Table S2: All the identified, quantified, and deregulated proteins at different radiation doses compared to the sham-irradiated control at 24 h; Table S3: All the identified, quantified, and deregulated proteins at the different radiation doses compared to the sham-irradiated control at 48 h; Table S4: All the identified, quantified, and deregulated proteins at different radiation doses compared to the sham-irradiated control at one week; Table S5: IPA pathway analysis and molecular functions for the radiation effect at one week, Table S6: All the identified, quantified, and deregulated proteins at different time points compared to the 4 h time point in the sham-irradiated control; Table S7: All the identified, quantified, and deregulated proteins at the different time points compared to the 4 h time point in 0.25 Gy irradiated samples; Table S8: All the identified, quantified, and deregulated proteins at the different time points compared to the 4 h time point in the 0.5 Gy irradiated samples; Table S9: All the identified, quantified, and deregulated proteins at the different time points compared to the 4 h time point in the 2 Gy irradiated samples; Table S10: All the identified, quantified, and deregulated proteins at different time points compared to the 4 h time point in 10 Gy irradiated samples; Table S11: Ingenuity pathway analysis and molecular functions for each time and dose point.
